# Chloral Hydrate

**Published:** 1875-09

**Authors:** 


Chloral Hydrate.—On this body Professor Konig writes as follows: The narcotic power of chloral hydrate is increased to an important extent, and its prejudicial effects reduced in the same proportion, if bicarbonate of sodium be administered immediately before. After five observations, I believe myself to be in a position to state that one part, by weight, of chloral hydrate, may be replaced, with equal effect by one part of bicarbonate of sodium and four-tenths of chloral hydrate.—*Medical and Surgical Reporter.*

